# Surface passivation of intensely luminescent all-inorganic nanocrystals and their direct optical patterning

**DOI:** 10.1038/s41467-022-35702-7

**Published:** 2023-01-04

**Authors:** Pengwei Xiao, Zhoufan Zhang, Junjun Ge, Yalei Deng, Xufeng Chen, Jian-Rong Zhang, Zhengtao Deng, Yu Kambe, Dmitri V. Talapin, Yuanyuan Wang

**Affiliations:** 1 State Key Laboratory of Coordination Chemistry, School of Chemistry and Chemical Engineering, Nanjing University, 210023 Nanjing, China; 2 College of Engineering and Applied Sciences, Nanjing University, 210023 Nanjing, China; 3 NanoPattern Technologies, Inc., Chicago, IL 60637 USA; 4 Department of Chemistry and James Franck Institute, University of Chicago, Chicago, IL 60637 USA

**Keywords:** Quantum dots, Quantum dots

## Abstract

All-inorganic nanocrystals (NCs) are of great importance in a range of electronic devices. However, current all-inorganic NCs suffer from limitations in their optical properties, such as low fluorescence efficiencies. Here, we develop a general surface treatment strategy to obtain intensely luminescent all-inorganic NCs (ILANs) by using designed metal salts with noncoordinating anions that play a dual role in the surface treatment process: (i) removing the original organic ligands and (ii) binding to unpassivated Lewis basic sites to preserve the photoluminescent (PL) properties of the NCs. The absolute photoluminescence quantum yields (PLQYs) of red-emitting CdSe/ZnS NCs, green-emitting CdSe/CdZnSeS/ZnS NCs and blue-emitting CdZnS/ZnS NCs in polar solvents are 97%, 80% and 72%, respectively. Further study reveals that the passivated Lewis basic sites of ILANs by metal cations boost the efficiency of radiative recombination of electron-hole pairs. While the passivation of Lewis basic sites leads to a high PLQY of ILANs, the exposed Lewis acidic sites provide the possibility for in situ tuning of the functions of NCs, creating opportunities for direct optical patterning of functional NCs with high resolution.

## Abstract (web summary)

All-inorganic nanocrystals are of great importance for a variety of electronic applications. Here, the authors use metal salts to remove organic ligands to obtain passivated nanocrystals with improved fluorescence yield for direct optical patterning.

## Introduction

Over the past decades, colloidal nanocrystals (NCs), especially quantum dots (QDs), have emerged as a versatile class of functional building blocks for electronic^1^, optoelectronic^2–4^, catalytic^5–7^, and bioimaging applications^8^ due to their well-controlled compositions and size-tunable optoelectronic properties combined with low-cost solution processability^9^. Semiconductor NCs, synthesized by conventional colloidal methods, are often capped with long-chain organic ligands, which determine NC growth and provide NC stability in nonpolar solvents. Because of the passivation of the dangling bond on the NC surface, organically capped NCs show excellent photoluminescence (PL) efficiency. However, organic ligands with long hydrocarbon chains greatly hinder charge transport between NCs^10,11^.

Inorganic ligands can be used to dramatically improve charge transport in NC layers. This concept was first introduced by Talapin’s group in 2009, where inorganic molecular metal chalcogenide complexes replaced bulky organic ligands and provided colloidal stability of individual NCs in polar systems^12^. Later, several families of inorganic ligands (halides, halometalates, chalcogenides, oxometalates, polyoxometalates, etc.) were discovered to broaden the scope of ligand functionality and to expand the chemical versatility of colloidal all-inorganic NCs^13–15^. These achievements in all-inorganic NCs enabled a higher packing density of NC films and higher electron mobility, thus promoting the performance of NC electronic devices. However, obtaining reliable all-inorganic NCs with fewer surface defects is difficult^13,16^. Surface modification with existing inorganic ligands inevitably generates defects at the surface of NCs, leading to a dramatic decrease in photoluminescence quantum yield (PLQY)^13,17,18^.

In this work, we introduce a general metal salt treatment to obtain intensely luminescent all-inorganic NCs (ILANs). The metal salts used for ligand exchange consist of metal cations (Cd^2+^, Zn^2+^, Pb^2+^, and In^3+^) and noncoordinating anions (NO_3_^−^, BF_4_^−^, or triflate OTf^−^). In comparison with previous inorganic ligands, one feature of these metal salts is that they played several roles in the surface treatment process: (i) gently removing the original organic ligands and providing colloidal stability of NCs in polar solvents, (ii) binding to unpassivated Lewis basic sites to preserve the luminescent properties of NCs and (iii) the exposed metal sites enable NC surface functionalization by more complex capping molecules with programmed functions. As a result, the NC after treatment exhibited excellent PL efficiency. The PLQYs of red-, green- and blue-emitting ILANs achieved 97%, 80%, and 72% in DMF, respectively. For reference, the original NCs capped with native organic ligands showed PLQYs of 97%, 84%, and 82% for the red-, green- and blue-emitting NCs used in this study.

To explore the reasons for the high PL efficiency, we studied the surface environment of ILANs through time-resolved PL decay and electrochemiluminescence (ECL) spectroscopy. We demonstrated that compared with other inorganic ligand surface treatment methods, metal salt treatment can greatly reduce the number of surface traps. A quantitative analysis via inductively coupled plasma optical emission spectroscopy (ICP-OES) indicated that the metal salt was indeed bound to the NC surface. Further studies by NMR confirmed that the binding between metal salts and Lewis basic sites on the NC surface resulted in better passivation of all-inorganic NCs and higher PLQY of ILANs. The exposed Lewis acidic sites on the ILAN surface can be further modified with delicately designed capping ligands. We showed that after systematic surface property engineering, ILANs were suitable for various industrially adapted patterning techniques, including direct optical lithography of functional inorganic nanomaterials (DOLFIN)^19,20^ and inkjet printing, to design luminescent microscale structures with a spatial resolution down to 2 μm and a line edge roughness of 200 nm. Recently, many NC patterning strategies have been used to fabricate QLEDs with high performance^21–23^. The ability to reliably obtain ILANs along with the development of various material-adapted patterning techniques creates a versatile platform for next-generation QD-based optoelectronic devices.

## Results and discussion

**Fig. 1:**
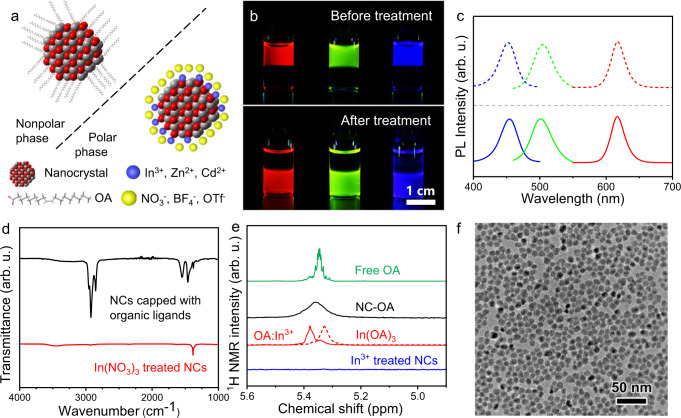
Formation of ILANs. **a** Scheme of the surface treatment of NCs with M(NO_3_)_*x*_, M(OTf)_*x*_, and M(BF_4_)_*x*_. **b** Optical image of red-emitting CdSe/ZnS NCs, green-emitting CdSe/CdZnSeS/ZnS NCs, and blue-emitting CdZnS/ZnS NCs before and after treatment with In(NO_3_)_3_. The solvents of the upper layer and bottom layer were hexane and DMF, respectively. **c** PL spectra of red-, green- and blue-emitting Cd-based NCs before (dashed lines) and after (solid lines) surface treatment. **d** FTIR spectra of OA-capped and In(NO_3_)_3_-treated CdSe/CdZnSeS/ZnS NCs. **e** ^1^HNMR spectra of free OA (green curve), OA-capped CdSe/CdZnSeS/ZnS NCs (NC-OA, black curve), In(OA)_3_ (red dots), OA:In^3+^ complexes (red curve) and In(NO_3_)_3_-treated CdSe/CdZnSeS/ZnS NCs (blue curve). **f** TEM images of In(NO_3_)_3_-treated CdSe/CdZnSeS/ZnS NCs.

Core–shell NCs with compositions of CdSe/ZnS, CdSe/CdZnSeS/ZnS, and CdZnS/ZnS were chosen as red-, green- and blue-emitting Cd-based NCs, respectively. The red and green-emitting InP/ZnSeS/ZnS NCs were composed of InP NCs with different core sizes and epitaxially grown ZnSeS/ZnS shells. Both Cd-based and In-based core–shell NCs were synthesized following previously reported procedures using oleic acid (OA) as a surface ligand and dissolved in nonpolar solvents (hexane, toluene, etc.) as a colloidal solution^24–27^. The PLQYs of the red-, green-, and blue-emitting Cd-based NCs were 97%, 84%, and 82%, respectively, which are listed in Supplementary Table 1 together with In-based NCs with QYs of 66% (red) and 76% (green). ILANs were obtained through a ligand exchange procedure, which is described in the “Methods” section. During the ligand exchange procedure, metal salts stripped the organic ligands from the NC surface, forming cationic bare NCs stabilized in polar solvents (Fig. 1a, b). In a typical ligand exchange experiment, a hexane solution containing organically capped NCs was added to a DMF solution containing metal salts to form a two-phase mixture. After vigorous stirring, a complete phase transfer of NCs from hexane to DMF was observed (Fig. 1b). Additionally, a one-phase method is available for the ligand exchange. A DMF solution containing metal salts was added to an NC toluene solution, forming a homogeneous phase. When the exchange process was completed, NCs precipitated and could be separated through centrifugation. Most of the ILANs in the following discussions were prepared via the one-phase method.

### Formation of intensely luminescent all-inorganic NCs

According to the hard–soft acid–base (HSAB) principle, hard Lewis acids bind more tightly to hard Lewis bases. The driving force of the surface ligand exchange is the stronger binding affinity of metal salt cations to organic ligands. Since OA is hard, the binding affinity of metal ions to OA followed the order of In^3+^ > Zn^2+^ > Cd^2+^ according to their hardness order. Therefore, Zn^2+^ can strip OA from CdSe NCs but not InP NCs. We should note that for compound NCs, the hardness of metal ion sites at the NC surface can be reduced by the interaction with other soft atoms inside the NCs^10,13^. As a result, all the metal salts composed of cations of In^3+^, Zn^2+^, and Cd^2+^ can strip OA from CdSe NCs, and In^3+^ can achieve such exchange on InP NCs (Supplementary Fig. 1). Table 1 lists the NCs, original surface ligands and corresponding cations of metal salts used for ligand exchange in this work. The resulting colloidal solutions of all-inorganic NCs ligand-exchanged and colloidally stabilized with bare cations are shown in Supplementary Fig. 2.

**Table 1:** Overview of NCs, surface ligands, and corresponding cations of metal salts used for ligand exchange

NCs	NC types	Original ligands	Cations
CdSe	Dots	OA	Cd^2+^, Zn^2+^, In^3+^
CdSe	Dots	ODPA	In^3+^
ZnSe	Dots	OA	Zn^2+^, In^3+^
PbS	Dots	OA	Cd^2+^, Zn^2+^, In^3+^
InP	Dots	PA	In^3+^
TiO_2_	Dots	OA/OLA	Cd^2+^, Zn^2+^, In^3+^
FePt	Dots	OA/OLA	Cd^2+^, Zn^2+^, In^3+^
CdSe/ZnS	Dots	OA	Zn^2+^, In^3+^
CdSe/CdZnSeS/ZnS	Dots	OA	Zn^2+^, In^3+^
CdZnS/ZnS	Dots	OA	Zn^2+^, In^3+^
InP/ZnSeS/ZnS	Dots	OA	Zn^2+^, In^3+^
CdSe	Wires	OA	Zn^2+^, In^3+^
CdSe (wz)	Platelets	OCA	Cd^2+^, Zn^2+^, In^3+^
CdSe (zb)	Platelets	MA	Zn^2+^, In^3+^

*OA* oleate, *OLA* oleylamine, *OCA* octylamine, *MA* myristate, *PA* palmitate, *ODPA* octadecylphosphonate; anions: OTf^−^, NO_3_^−^, BF_4_^−^.

The resulting all-inorganic core–shell NCs showed an intensely luminescent property similar to that of the pristine NCs (Fig. 1c) and were thus named ILANs. A few characterizations shown in Fig. 1 and Supplementary Fig. 4 compared the features of ILANs obtained from In(NO_3_)_3_ treatment and pristine red-, green- and blue-emitting NCs capped with oleate (OA) ligands. In the FTIR spectra of CdSe/CdZnSeS/ZnS NCs (Fig. 1d), the vibrational peaks at 2800–3000 and 1400–1650 cm^−1^ belong to the C–H bonds of the OA surface ligand of the pristine NCs. After treatment, these peaks completely disappeared, indicating that the NCs were converted into all-inorganic NCs. The remaining single peak at 1380 cm^−1^ corresponded to the vibration of N–O bonds in NO_3_^−^ anions. It should be noted that NO_3_^−^ anions, as well as BF_4_^−^ and OTf^−^, acted as counterions rather than bonding ligands since they were not able to coordinate with surface metal atoms tightly^28^. The counterions can form a diffuse double layer to stabilize the NCs in polar solvents (Supplementary Fig. 3)^10^. Since the surface metal cations were not directly capped by any ligands, we referred to the resulting NCs as “cationic bare” NCs.

We also confirmed the stripping of organic ligands through ^1^H NMR spectroscopy. The ^1^H NMR signal of protons of the double bond in OA observed at 5.3–5.4 ppm can be generally used to distinguish between free and surface-bound molecules, with free molecules showing sharp NMR peaks and molecules bound to the NC surface characterized by broadened NMR peaks (Fig. 1e, green curve and black curve)^29^. A broad signal was not observed after surface treatment (Fig. 1e, blue curve), indicating that OA was fully stripped. To further investigate the entire process of the surface reaction, we precipitated the resulting ILANs by adding toluene and measured the ^1^H NMR spectrum of the supernatant. The center of the broad signal shifted downfield from 5.35 to 5.38 ppm, and the line width was reduced (Fig. 1e, red curve). Meanwhile, a shoulder peak at ~5.34 ppm occurred. The reduced line width was different from both free and surface-bound OA. We attributed the signal to In(OA)_*x*_ (1 < *x* < 3), generated by the combination of In^3+^ and OA, because the ^1^H NMR signal of In(OA)_3_ showed a similar line width and position (Fig. 1e, red dots). It should be noted that *x* was not assigned to be a fixed value; otherwise, the signal should be single without any shoulder peaks. Since there were two overlapping signals, we believed that each added In^3+^ may take away one, two, or three OA molecules from the NC surface. A similar phenomenon was also observed by Helms and coworkers in a previous report^30^.

The exposure of uncoordinated acidic sites on the ILAN surface resulted in a positively charged surface, leading to a ζ-potential of +25 mV (Supplementary Fig. 4a), which was sufficient for the electrostatic stabilization of NCs in polar solvents with a concentration up to 100 mg mL^−1^ ^10^. The colloidal stability was verified by the flat curve in small angle X-ray scattering (SAXS, Supplementary Fig. 4b). As shown in the TEM images (Fig. 1f and Supplementary Fig. 4c), the morphology was retained after surface treatment. The diameters of the OA-capped and In(NO_3_)_3_-treated CdSe/ZnS NCs were both 12 nm, which proved that no etching occurred. Resulting from the removal of long-chain ligands, a significant decrease in edge-to-edge distance between NCs from 2–3 nm to nearly zero can be observed clearly. In addition to the interparticle distance, the work function of NC solids was also affected by the surface treatment. The work function of CdSe/CdZnSeS/ZnS NCs was increased by 0.16 eV, which was calculated via the secondary electron cutoff regions in ultraviolet photoelectron spectroscopy (UPS) (Supplementary Fig. 4d). We attributed the difference to the changes in the surface dipole, which is determined by the dipole moment at the surface–ligand interface and the ligand molecular structures^10^. Therefore, the use of metal salt treatment provided a step to adjust the work function of colloidal NCs^31^. The removal of insulating organic layers and shortening interparticle distances help to enhance the electronic exchange coupling energy in NC solids and increase their packing density^12^. This makes metal-salt-treated ILANs promising for the fabrication of NC-based optoelectronic devices.

**Fig. 2:**
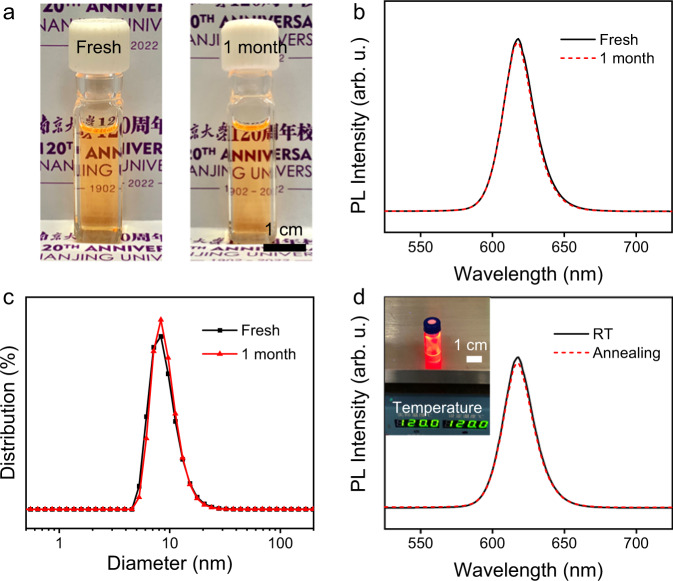
Long-term and thermal stability of ILANs. **a** CdSe/ZnS ILANs in DMF stored under ambient conditions for one week and one month. **b** PL spectra of CdSe/ZnS ILANs before and after storage (without normalization). **c** Particle size distribution of CdSe/ZnS ILANs before and after storage, measured by dynamic light scattering (DLS). **d** PL spectra of CdSe/ZnS ILANs before and after heating on a hot plate at 120 °C for 30 min. Inset: CdSe/ZnS ILANs on a hot plate under UV light.

The long-term stability and thermal stability of ILANs were tested. An ILAN sample in DMF was prepared and stored under ambient conditions (air, room temperature, and room light). It is stable for a couple of weeks (Fig. 2a). The particle size and optical properties were not changed, as evidenced by the DLS and PL spectral measurements of the ILANs after one month of storage (Fig. 2b, c). For thermal stability, we heated an ILAN sample in DMF with a hot plate at 120 °C for 30 min; afterward, it remained as a colloid, and PL was not changed as shown in Fig. 2d.

### Optical properties of ILANs and related surface chemistry

NC-based optoelectronic devices require a combination of good electronic properties and optical properties. However, all-inorganic NCs developed in recent years showed impressive electronic properties (e.g., high charge mobility), while their optical properties suffered due to surface traps generated from the ligand exchange process^12,14,32^. In this section, we focus on the study of the optical properties of ILANs.

**Fig. 3:**
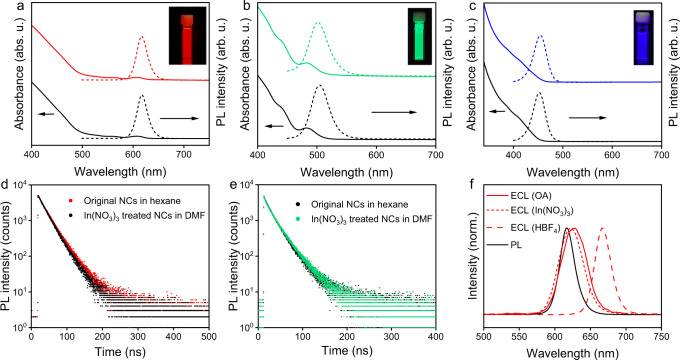
Optical properties of the all-inorganic NCs. **a**–**c** Absorption and emission spectra of CdSe/ZnS, CdSe/CdZnSeS/ZnS, and CdZnS/ZnS NCs before (black lines) and after (colorful lines) the surface treatment. **d** and **e** Time-resolved PL decay profiles of CdSe/ZnS NCs and CdSe/CdZnSeS/ZnS NCs before and after the surface treatment. **f** Comparison of the PL and ECL spectra of CdSe/ZnS NCs before and after surface treatment.

To study the optical properties, both the absorption and emission spectra of the ILANs were measured and compared with those of the original NCs (Fig. 3a–c). Generally, the shift of excitonic features in absorption spectra suggests size changes in NCs. After surface treatment with In(NO_3_)_3_, the excitonic features of the red-, green- and blue-emitting ILANs in the absorption spectra were all unchanged, indicating a preserved shape and size. However, due to the gradient-alloy structures of such core–shell NCs, their excitonic features are not very distinct in absorption spectra^33^. Therefore, one may argue that even if there is a change, it might not be observable for core–shell NCs. To draw a more reliable conclusion, we used wurtzite (wz) CdSe NCs with distinct characteristic absorption peaks as a model system and monitored the changes in the absorption spectra (Supplementary Fig. 5). Our results indicated that regardless of the metal salts we used, the original structured absorption features of CdSe NCs were retained. The clear and fixed excitonic features proved that no etching or shell growth occurred during surface treatment.

It has been shown that the removal of organic ligands can form surface dangling bonds, resulting in electronic trap states and a decrease in the PLQY^34^. Here, we demonstrated that despite the loss of surface passivation from organic ligands, both Cd-based and In-based core–shell ILANs preserved efficient luminescence (Supplementary Table 1). The QYs of red-emitting CdSe/ZnS ILANs and green-emitting CdSe/CdZnSeS/ZnS ILANs were 97% and 80%, respectively, which were nearly equal to those of the pristine NCs. The treatment of CdSe/ZnS NCs with In(NO_3_)_3_, In(BF_4_)_3_, and In(OTf)_3_ gave the same result for PL efficiency (Supplementary Fig. 6). InP NCs, as a potentially less toxic alternative to Cd-based NCs, have attracted considerable interest, yet luminescent all-inorganic InP NCs have rarely been reported. We demonstrated that green- and red-emitting InP/ZnSeS/ZnS NCs were successfully converted into ILANs with PLQYs of 57% and 39%, respectively (Supplementary Fig. 7, Supplementary Table 1). The slightly lower PLQY of In-based ILANs compared with that of Cd-based ILANs can be attributed to the NC surface being more sensitive to the environment and the insufficient shell coverage caused by lattice mismatch^27^. An improved PL performance of In-based ILANs should be expected when In-based NCs with a uniform thicker shell is used. More surprisingly, blue-emitting CdZnS/ZnS ILANs, whose surface was not as stable as the surface of red or green NCs, achieved a PLQY of 72%, only 12% lower than that of the original NCs. In comparison, the PL of the same blue-emitting NCs was almost fully quenched after treatment with other as-reported inorganic ligands (Supplementary Fig. 8a). As control experiments, typical X-type inorganic ligands were compared with In(NO_3_)_3_ surface treatment. The PLQY of green-emitting NCs decreased to only ~30% and ~15% of the pristine NCs after ligand exchange with thiocyanate and ZnCl_4_^2−^, respectively. We further used titrations to monitor the evolution of PL intensities as a function of the concentration of foreign inorganic ligands and introduced the Stern–Volmer (S–V) ratio to evaluate the effects of the corresponding treatment^35^. As titration progressed, the PL of green-emitting CdSe/CdZnSeS/ZnS NCs decreased dramatically despite the low concentration of conventional inorganic ligands, while the PL of In(NO_3_)_3_-treated NCs did not change significantly with increasing metal salt concentration (Supplementary Fig. 8b). In addition, the PL should be affected if the concentration of metal salts was too high (Supplementary Fig. 9) since the high acidity of the salt solution would damage the NC surface. The PL spectra and PLQY of ILANs in the form of the film were also measured. Coated on the quartz substrate, the PL spectra of ILANs were not changed (Supplementary Fig. 10). The PLQY slightly decreased, which was influenced by the shortened inter-particle distance (Supplementary Table 2).

All-inorganic NCs can be obtained by treating the pristine NCs with various inorganic ligands. Surprisingly, however, only metal salt-treated NCs in this work show intense luminescence. This result inspired us to further study the related surface chemistry during the ligand exchange process. Time-resolved PL measurements were performed to investigate time-resolved carrier dynamics in ILANs before and after ligand exchange. Figure 3d shows the time-resolved PL decays of red-emitting CdSe/ZnS NCs before and after treatment with In(NO_3_)_3_. Both curves can be accurately fitted with first-order exponential functions (goodness-of-fit *R*^2^ > 0.999), indicating that no additional nonradiative recombination pathway occurred in the ILANs. The PL lifetime for ILANs was calculated to be 24 ns from the PL decay curves, which was the same as that of pristine NCs (Supplementary Table 1). In addition to red-emitting ILAN, green-emitting CdSe/CdZnSeS/ZnS ILAN also showed unchanged PL kinetics and lifetimes (Fig. 3e). These results of Cd-based red and green ILANs indicated that metal salt-treated NCs have fewer surface trap states than other inorganically functionalized NCs. Slightly different from the red and green ILANs, surface treatment of blue-emitting CdZnS/ZnS NCs with In(NO_3_)_3_ caused a decrease in the PL lifetime, perhaps due to the formation of a nonradiative trap pathway (Supplementary Fig. 8c). To further analyze the trend of surface traps induced by various ligands, the PL lifetimes of NCs treated with In(NO_3_)_3_, NOBF_4_ and Et_3_OBF_4_ were compared through time-resolved PL measurements. The PL kinetics of the green NCs changed from first-order exponential to second-order exponential when using NOBF_4_ or Et_3_OBF_4_ (Supplementary Fig. 8d), indicating the generation of a nonradiative pathway in the NCs. Therefore, all these traditional inorganic ligands introduce mid-gap trap states during surface treatment, but metal salts do not.

To further investigate the surface trap of ILANs, electrochemical luminescence (ECL), generated by electrochemical injection of holes into the 1S(h) state of NCs following electrochemical reduction of certain peroxide compounds, was introduced^36^. According to previous reports, the ECL spectra can be more sensitive to the surface environment of NCs than PL, which is dominated by excitation and emission within the core^37^. The ECL spectra show an obvious redshift from the PL spectra when NCs have significant surface traps, which can be eliminated if the NC surface is sufficiently passivated^38,39^, We chose K_2_S_2_O_8_ as the ECL co-reactant, and the ECL spectra were measured at −2.0 V (vs. Ag/AgCl), where NCs and S_2_O_8_^2−^ absorbed electrons and then generated excited-state NCs for light emission^40^. Compared with the PL emission band, the central wavelength in the ECL spectra of red NCs shifted ~7 nm, and the position was almost retained after the original NCs were converted to ILANs (Fig. 3f). The difference between the PL and ECL emission wavelengths can be attributed to the inner-filter effect^41–43^. The ECL band did not redshift after the surface treatment, suggesting that a minimum amount of surface traps was generated, which was consistent with the aforementioned PL decay results. The ECL intensity of ILANs, measured by photomultiplier (PMT) while scanning with a triangle wave (−2 to 0 V, vs. Ag/AgCl), was slightly lower than that of the pristine NCs (Supplementary Fig. 11). As a control experiment, the protic acid HBF_4_ was employed to treat OA-capped red-emitting NCs (Fig. 3f). A large redshift of the ECL spectrum was observed, indicating that many surface trap states were generated due to the harsh surface treatment.

### Relationship between the ligand binding mechanism and PL

Since the luminescent properties of NCs are related to their surface dangling bonds^34^, cationic bare NCs may be prone to have more trap states than NCs capped with organic ligands. However, the studies of time-resolved PL and ECL illustrated that such surface traps were largely absent in cationic bare ILANs. Thus, we attribute the high PL performance of ILANs to the unique passivation effect of metal salts. The surface trap states of binary semiconductor NCs (II–VI, III–V, and IV–VI) can be introduced by the exposed undercoordinated chalcogenide sites (E) with dangling bonds^44–46^. Therefore, we propose that for ILANs, passivation occurred at E sites through binding between metal salts and the NC surface.

**Fig. 4:**
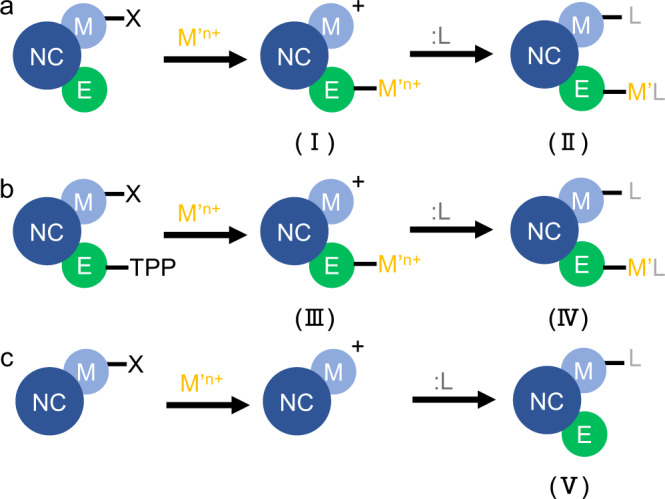
Schematic illustration of surface treatment processes. **a** NCs capped with X-type organic ligands were treated with metal salts (Sample I) and subsequently modified by L-type organic ligands (Sample II). **b** NCs capped with both X-type organic ligands and TPP were treated with metal salts (Sample III) and subsequently modified by L-type organic ligands (Sample V). **c** NCs with metal atoms occupying the majority of surface area (NPLs) were treated with metal salts and subsequently modified by L-type organic ligands (Sample V).

To verify this hypothesis, we first used ICP-OES measurements to monitor the surface treatment process on CdSe/CdZnSeS/ZnS core–shell NCs. ILANs was first obtained in the polar phase with In(NO_3_)_3_ treatment (Sample I in Fig. 4a) and then subsequently transferred back to the nonpolar phase by secondary surface modification with oleylamine (OLA) to preclude any free In^3+^ from detection (Sample II). Surprisingly, the same amount of In^3+^ cations (~26% vs. Cd) was detected in both samples, indicating that the metal salts were indeed bound at the NC surface (Fig. 4a). Similarly, indium was found by elemental mapping through TEM (Supplementary Fig. 12). Next, we investigated the interaction between metal salts and the NC surface. The surface of binary nanocrystals is composed of metal (M) atoms and nonmetal (E) atoms, both of which can be bonded to surface ligands^10,34^. Since the binding of inorganic ligands to the NC surface usually occurs at Lewis acidic sites (i.e., M sites), we first used zinc blende (zb) CdSe nanoplatelets (NPLs), where Cd atoms occupy the majority of the surface area^47^, as a model system to investigate the possibility of binding of metal salts to M sites on the NC surface. As shown in Table 2, the absence of In ions in the treated zb CdSe NPLs indicated that the metal cations did not bind with the Lewis acidic sites on the NPL surface (Fig. 4c, Sample V). We cannot rule out the possibility that the metal salts were bound to the side facets of zb CdSe NPLs (either true (−1−10) facets or a combination of (111) facets and (−111) facets)^48^. However, since these facets are a minority in zb CdSe NPLs, the bound metal salts, even if real, are present in quantities below the detection limit of the instrument and cannot be detected.

**Table 2:** ICP–OES results of NCs treated following the steps in Fig. 4

Sample	Treatment	[Cd] (µM)	[In] (µM)	[In]/[Cd]
I	Core–shell NCs + In	52.8	14.1	0.267
II	Core–shell NCs + In + L	50.6	13.4	0.264
III	Core–shell NCs + TPP + In	65.4	17.3	0.265
IV	Core–shell NCs + TPP + In + L	50.8	13.6	0.268
V	zb-CdSe NPLs + In + L	14.0	ND	ND

Core–shell NCs were CdSe/CdZnSeS/ZnS NCs exposing both M and E sites. zb CdSe NPLs approximately represent NCs exposing only M sites. L stands oleylamine as an L-type ligand.

*ND* not detected, or quantities are below the detection limit of the instrument.

**Fig. 5:**
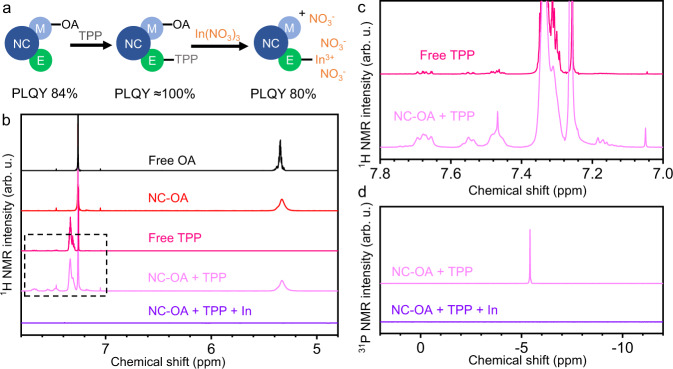
Surface treatment on TPP-modified NCs. **a** Scheme of TPP and In^3+^ treatment of CdSe/CdZnSeS/ZnS NCs capped with OA and the PLQY of each stage. **b** ^1^HNMR of free OA, OA-capped NCs, free TPP, TPP-modified NCs, and In^3+^-treated NCs. The dashed-frame area is magnified in (**c**). **d** ^31^PNMR of TPP-modified NCs and In^3+^-treated NCs.

Together with the above experimental results, we proposed that incoming metal cations may bind with E sites exposed at the NC surface, resulting in intense luminescence of ILANs. To further test this hypothesis, we treated triphenylphosphine (TPP)-capped NCs with In(NO_3_)_3_ and monitored the changes in PLQY (see scheme in Fig. 5a). TPP can weakly bind with surface E sites and passivate the NC surface traps^49^. Adding excess TPP to OA-capped CdSe/CdZnSeS/ZnS NCs resulted in an increasing PLQY to nearly 100%, indicating that TPP successfully passivated NCs through weak binding. Figure 5b describes the ^1^H NMR results of OA-capped NCs and TPP-modified NCs. The broad signal at 5.3 ppm suggested that OA was not dissociated from the ZnS shell after TPP modification, while new signals at 7.0–7.8 ppm, which were assigned to TPP, appeared and grew in intensity (Fig. 5c), both of which demonstrated that TPP bound to the S sites of the ZnS shell. This was consistent with a previous report^27^. After introducing In(NO_3_)_3_ into TPP-modified NCs, ILANs were obtained as expected. The disappearance of all the ^1^H NMR and ^31^P NMR signals as shown in Fig. 5b and d, demonstrated that In(NO_3_)_3_ not only stripped OA from the NCs but also substituted the TPP bound to the S site (Fig. 4b), which was also evidenced by the presence of the same amount of In^3+^ illustrated from the ICP-OES data (Samples III and IV in Table 2). The resulting PLQY was 80%, which was similar to that of the NCs directly treated with In(NO_3_)_3_ (Fig. 5a). Therefore, we believe that the added cations play dual roles: (i) ligand stripping and (ii) binding and electronic passivation of Lewis basic surface sites. The complete chemical equation can be written as

1 $$\begin{array}{c}{n}[{({{{{{\rm{ME}}}}}})}_{{a}}{({{{{{{\rm{MX}}}}}}}_{{m}})}_{{b}}]+({bm}+{cn})({{{{{\rm{M}}}}}}{\hbox{'}} {{{{{{\rm{Y}}}}}}}_{{n}})\to \\ {n}{[{({{{{{\rm{ME}}}}}})}_{{a}}{{{{{{\rm{M}}}}}}}_{{b}}{{{{{\rm{M}}}}}}{\hbox{'}}_{{c}}]}^{({bm}+{cn})+}\cdots ({bm}+{cn}){{{{{{\rm{Y}}}}}}}^{-}+{bm}({{{{{\rm{M}}}}}}{\hbox{'}} {{{{{{\rm{X}}}}}}}_{{n}}),\end{array}$$

where (ME)_*a*_(MX_*m*_)_*b*_ and M’Y_*n*_ represent the original NCs and added metal salts, respectively. During the surface treatment, some metal cations remove the strongly bound ligands (*bm* mole of M’ in Eq. (1)), and the others become new coordinated ligands for surface healing (*cn* mole of M’ in Eq. (1)).

Organic ligands and z-type ligands are able to passivate the NC surface in nonpolar solvents^34,46^. Herein, we showed that the binding of metal cations can also play the role of surface ligands and partially passivate electronic trap states by eliminating the surface dangling bonds. The resulting ILANs exhibited high colloidal stability in polar solvents, and the absorption spectra of wz CdSe NCs were identical before and after Cd^2+^, Zn^2+^, and In^3+^ salt treatment (Supplementary Fig. 5), indicating that the metal salts used in this work are different from traditional ligands and have their own uniqueness.

### Patterning ILANs by optical lithography and inkjet printing

One feature of ILANs is that the Lewis basic sites on the NC surface are passivated by metal salts, while the Lewis acidic sites are exposed, leading the ILANs to be cationic bare NCs. Utilizing the undercoordinated metal atoms (Lewis acid sites) as bridges for the surface functionalization of NCs with programmed functions, we turned ILANs into patternable inks and used the DOLFIN technique to directly pattern highly luminescent NCs^19,20,50^. To implement the DOLFIN process, photoamine generators, named PAmGs, were added to the ILAN colloids to form colloidal stable photosensitive inks. The PAmGs were synthesized by condensing coumaric acid and alkylamine under ambient conditions. For example, PAmG-BTA (*n*-butylamine) was isolated by mixing coumaric acid with *n*-butylamine and showed a broad absorption band at UV region (Supplementary Fig. 13)^51^. Upon exposure to deep UV, PAmG-BTA molecules decomposed and released *n*-butylamine, which can be expressed in Supplementary Fig. 14.

**Fig. 6:**
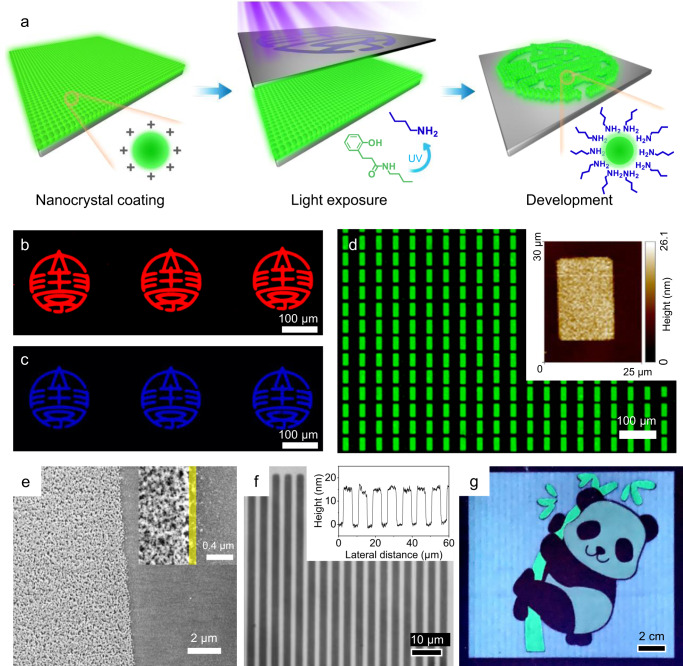
Patterning of all-inorganic NCs. **a** Schematic illustration of PAmG-assisted DOLFIN. **b**–**d** Optical images of CdSe/ZnS, CdZnS/ZnS, and CdSe/CdZnSeS/ZnS NC patterns via PAmG-assisted DOLFIN. Inset: AFM height image of a single pixel. **e** SEM image of the edge of the patterns. Inset: the boundary region, highlighted with a yellow band. **f** Optical microscopy images of patterned CdSe NCs with a line width of 2 μm. Inset: height profile of line-space features. **g** Inkjet-printed multicolor NC patterns under UV light.

The released primary amines subsequently bound to exposed surface cations of NCs, thereby reducing the solubility of NCs in DMF or NMF and forming uniform patterns with a resolution approaching the limit of the photomasks (Fig. 6a).

In addition to tuning the solubility of NCs, released primary amines play two other important roles that make the approach unique when compared to other variations of direct optical lithography techniques: (i) amines electronically passivate the NC surface and provide high PLQY and (ii) amine-capped NCs are more electrochemically stable than NCs capped with carboxylate ligands^11^. The exposure dose for patterning these RGB NCs can achieve ~100 mJ cm^−2^, which is at the same level as traditional photoresist-based lithography (e.g., for reference, Shipley S1800 series photoresist requires comparable exposure doses of 80–150 mJ cm^−2^). Figure 6b–d shows luminescent images patterned with all-inorganic NCs mixed with PAmG-BTA. The AFM image of an individual pixel indicated a flat surface (Fig. 6d, inset), where the RMS was ~1.4 nm. The line edge roughness was determined from the SEM image, in which the edges of the patterned regions were sharp and clean with roughness below 200 nm (Fig. 6e). To test the resolution, we patterned 3.6-nm CdSe NCs using a line-space mask and easily achieved line widths of 2 μm (Fig. 6f). The thickness of the line-space features measured by the profilometer was ~15 nm, which was uniform in every feature. Thicker films can be obtained by controlling the ink concentrations and spin-coating parameters, which are listed in Supplementary Table 3. It should be noted that without optimization, the patterned ILAN film still preserved PLQY of 58%, which retained ~85% of the initial PLQY of ILAN film (Supplementary Table 2), suggesting that this method is competitive among resist-free patterning methods. In addition to core–shell ILANs, other ILANs, such as CdSe NCs, ZnSe NCs, PbS NCs, and InP/ZnS NCs, can also be direct optical patterned by PAmG-assisted DOLFIN, demonstrating the generality and versatility of this approach (Supplementary Fig. 15). It is worth noting that the dosages required for obtaining reliable patterns are slightly different, although the photosensitive molecules used are the same (Supplementary Table 4). This indicates that the sensitivity of the inks could be determined by the binding affinity between the molecules released from the PAmGs and the NC surface and should be optimized for each case.

In addition to photolithography, inkjet printing is also a general technique for patterning NCs due to the advantages of low cost and material adaptability^52^. The ILANs were secondarily modified with mercaptopropionate or citrate, making NCs easily dissolve in water without affecting their optical properties (Supplementary Fig. 16, the surface treatment process is described in SI as Secondary Surface Modification). To test the program-controlled patterning capability, we first printed each color individually (Supplementary Fig. 17). Then, as a proof of concept, we patterned a panda image with multicolor by tuning the component ratios of RGB NC inks (Fig. 6g). The background cyan color was obtained by mixing green- and blue-emitting NCs, and the white color on the facial area of the panda was prepared by mixing red-, green- and blue-emitting NCs in an appropriate proportion (Supplementary Fig. 18). The colloidal stability of the postmodified ILANs was not influenced after mixing with each other (Supplementary Fig. 18a, inset). In addition, we studied the resolution by printing characters with a width of ~1 mm, which is nearly the resolution limit of the ink-jet printer we used (Supplementary Fig. 19). Because of the convenience and quickness of patterning bright fluorescent nanomaterials on paper-like substrates, ILAN-based inkjet printing showed potential for anticounterfeiting and encryption applications^53,54^.

In summary, we designed metal salts composed of metal cations and noncoordinating anions to convert organically capped NCs into intensely luminescent cationic bare all-inorganic NCs. The resulting ILANs showed a high PLQY and preserved excitonic dynamics, indicating that the treatment generated no significant surface traps. Studying the mechanism of the surface treatment, we demonstrated that the metal salts both strip organic ligands from the NC surface and simultaneously passivate Lewis basic sites at the NC surface. To assemble the high-performance materials into designed structures for practical applications, ILANs were patterned through PAmG-assisted DOLFIN and inkjet printing. Because of their high PL performance and lack of organic ligands as insulating layers, we expect that ILANs could become building blocks for optoelectronic devices and have a wide range of applications.

## Methods

### Materials

The following chemicals were used as received: Cadmium oxide (CdO, 99.99%, Aladdin), zinc oxide (ZnO, 99.999%, Aladdin), Cd(OAc)_2_·H_2_O (99.99%, Aldrich), Zn(OAc)_2_·2H_2_O (99.99%, Aldrich), Cd(NO_3_)_2_·4H_2_O (99.99%, Aladdin), Zn(NO_3_)_2_·6H_2_O (99.99%, Aladdin), In(NO_3_)_3_·*x*H_2_O (99.99%, Alfa Aesar), Pb(NO3)_2_ (99.99%, Aladdin), ZnCl_2_ (99.95%, Alfa Aesar), selenium shots (99.999%, Aladdin), S pillows (99.999%, Alfa Aesar), ammonium thiocyanate (99.99%, Aldrich), trioctylphosphine (TOP, Strem, 97%), trioctylphosphine oxide (TOPO, 90%, Aladdin), tributylphosphine (TBP, 95%, Aladdin), triphenylphosphine (TPP, 99%, Aladdin), oleic acid (OA, 90%, Alfa Aesar), myristic acid (MA, 99%, Aldrich), n-butylamine (99%, Sinopharm), oleylamine (95%, Strem), n-octanethiol (98%, Aladdin), octadecene (ODE, 90%, Alfa Aesar), mercaptopropionic acid (MPA, 98%, Aladdin), and CdSe/ZnS core–shell NCs (5 mg mL^−1^ in octane, Najing Tech).

### Synthesis of CdSe NCs

Cadmium oleate (Cd(OA)_2_) stock solution was prepared by mixing CdO (643 mg, 5 mmol) with 10 mL of oleic acid (OA). After degassing at 150 °C, the flask was heated to 250 °C under dry N_2_. The suspension then became a colorless solution, indicating the formation of Cd(OA)_2_. Then degas at 150 °C again to scavenge the generating water and store it as a waxy solid. For the synthesis of wurtzite CdSe NCs, we mixed Cd(OA)_2_ stock solution (2.25 mL), TOPO (1.2 g), and ODE (12 mL), which was degassed at 100 °C under vacuum. The temperature was then increased to 300 °C under N_2_. At that point, a mixture containing 4 mL of TOPSe (1.0 M) and 3 mL of oleylamine was swiftly injected into the flask. The reaction was kept for 7 min and then cooled down to room temperature. EtOH was added to precipitate the resulting NCs, which were redissolved in toluene next. The washing process was repeated three times and the purified NCs were finally dissolved in 5 mL of toluene.

### Synthesis of CdSe/CdZnSeS/ZnS core–shell NCs

Zinc oleate (Zn(OA)_2_) stock solution was prepared by mixing 405 mg (5 mmol) ZnO with 10 mL of oleic acid (OA). After heating under vacuum at 120 °C, the flask was refilled with N_2_ and the temperature was raised to 240 °C. The suspension then became a light yellow solution, indicating the formation of Zn(OA)_2_. The heater was removed, and the flask was allowed to cool down to 100 °C. Then, 15 mL of ODE and 25 mL of oleylamine were added. The obtained solution was degassed at 120 °C and stored in a glovebox. For the formation of core–shell NCs, Zn(OA)_2_ (0.08 M) and 1-octanethiol (0.09 M) were synchronously injected into the hot CdZnSeS core mentioned above by a dual-channel syringe pump at a rate of 2.5 mL h^−1^ for one hour. The resulting CdZnSeS/ZnS NCs were washed with toluene/EtOH and finally redissolved in toluene.

### Synthesis of CdZnS/ZnS core–shell NCs

CdO (1 mmol), Zn(OAc) _2_·2H_2_O (5 mmol), OA (3.5 mL), and ODE (7.5 mL) were mixed in a three-neck flask. After degassing at 100 °C, the mixture was heated to 300 °C under nitrogen gas. 1 mmol of sulfur powder was quickly dissolved in ODE (1.5 mL) at ~100 °C and the solution was swiftly injected into the flask as soon as the temperature reached 300 °C. The reaction was kept at 310 °C for 8 min. Then, 1.5 mL of TBP containing 4 mmol of sulfur was injected for shell growth. The shell-growth step lasted for 40 min. The resulting CdZnS/ZnS NCs were washed with hexane/acetone and finally redissolved in hexane.

### Synthesis of zb CdSe NPLs

To prepare cadmium myristate [Cd(MA)_2_] as a precursor, 0.24 g NaOH and 1.37 g MA were fully dissolved in 240 ml methanol with 1 h of vigorous stirring. Then, 0.617 g of Cd(NO_3_)_2_·2H_2_O was dissolved in 40 ml of methanol and slowly added to the above solution. Subsequently, a white precipitate was obtained. The resulting precipitate was rinsed three times with methanol under vacuum filtration. Finally, it was transferred to a vacuum-drying oven at room temperature overnight. The resulting white product is Cd(MA)_2_. For the synthesis of NPLs, 240 mg of Cd(OAc)_2_·2H_2_O, 150 µL of OA, and 15 ml of ODE were degassed in a three-necked flask for 1 h at 80 °C. Then we raised the temperature to 180 °C and quickly injected 150 µL of TOP-Se (1 M). After washing three times with toluene, the products were dispersed in toluene.

### Synthesis of PAmG-BTA

A solution of EDC (1.05 g) in anhydrous DCM (40 mL) was added to a solution of trans-*o*-coumaric acid (0.90 g) in anhydrous THF (5 mL). *n*-Butylamine (0.49 mL) was then added to the mixture with stirring at room temperature. The mixture turned yellow immediately. After stirring overnight, water was added to quench the reaction and the organic layer was washed with 10% HCl, saturated NaHCO_3_, and water for each, followed by drying with sodium sulfate. After evaporating the solvent, chloroform was used for recrystallization to give PAmG-BTA as a white crystal. ^1^H NMR (400 MHz, d_6_-DMSO, δ ppm): 0.89 (3H, t), 1.32 (2H, m), 1.44 (2H, m), 3.17 (2H, m), 6.66 (1H, d), 6.82 (1H, t), 6.89 (1H, d), 7.17 (1H, t), 7.42 (1H, d), 7.63 (1H, d), 8.02 (1H, t).

### Surface treatment procedures

The NC surface treatment was achieved through both one-phase and two-phase systems. Because of the inter-solubility between DMF (or NMF) and toluene, no phase segregation takes place when adding the DMF solution to the toluene phase, leading to the one-phase system. If the two solvents are not inter-soluble, the surface treatment procedure will be accomplished by a two-phase approach. In a one-phase system, 1 mL of NC solution (2–10 mg mL^−1^ in toluene) was mixed with 100 µL of cation ligand solution (0.2–0.5 M). The resulting mixture was vigorously stirred or shaken until a precipitate of NCs was observed. After centrifugation, the precipitate was redissolved into polar solvents (DMF, NMF, DMSO, PC, etc.). The clear solution was precipitated one more time by adding toluene. The dried powder was then easily redispersed in pure polar solvents to form a stable solution with a high concentration (10–60 mg mL^−1^). In the two-phase system, 1 mL of NC hexane solution (2–10 mg mL^−1^) was added to a cation ligand solution (1 mL, 0.02–0.05 M in polar solvents) to form a two-phase mixture. After vigorous stirring, the hexane layer turned from dark to colorless, while the polar solvent layer turned to color, indicating that the NCs were completely transferred to the polar phase after ligand exchange. The NCs were then purified by hexane and precipitated from polar solvent by adding a nonsolvent such as acetonitrile or toluene. After centrifugation, NCs were redispersed in various polar solvents to form highly concentrated colloidal solutions (10–60 mg mL^−1^).

### Secondary surface modification

ILANs can be remodified by organic ligands through both a one-phase system and a two-phase system. In the one-phase system, 1 mL of ligand solution (0.02–0.1 M, toluene/hexane for OA or oleylamine, and water for MPA) was added to NCs precipitated from polar solvent by centrifugation. After vigorous stirring, remodified NCs were dispersed, forming a colloidal solution. EtOH was used to wash NCs one more time to remove free ligands. In the two-phase system, 1 mL of ligand solution (0.02–0.1 M in hexane) was mixed with 1 mL of NC colloidal solution (2–10 mg mL^−1^). After vigorous stirring, the NCs transferred into the hexane phase, indicating that the NCs were completely remodified by organic ligands. The resulting NC colloidal solution was washed one more time with EtOH.

### Characterizations

UV–Vis absorption spectroscopy was measured on an Agilent Cary 5000 spectrophotometer. The absolute PLQY was measured by using an instrument combining an integrating sphere and a computer-controlled spectrometer (Labsphere). Time-resolved PL was measured on a HORIBA FL-3 3D fluorescence spectrometer. Fourier transforms infrared (FTIR) spectra were acquired in the transmission mode or ATR mode using a Nicolet iS50 FTIR spectrometer. For NMR analysis, 5 mg of NC solids were dissolved in CDCl_3_ or d_6_-DMSO and the signals were recorded on a Bruker AVANCE III-500 spectrometer. Zeta potential was measured with a Malvern Nano-Z zeta-potential analyzer. A JEOL JEM-2800 transmission electron microscope (TEM) was employed for TEM investigations. To measure ECL spectroscopy, NCs were drop-coated on a Pt electrode. Pt wire and Ag/AgCl were the counter electrode and reference electrode, respectively. The electrolyte was 0.1 mol L^−1^ phosphate-buffered saline (PBS, pH = 7.4) containing 50 mmol L^−1^ K_2_S_2_O_8_. ECL spectra were measured at −2.0 V (vs. Ag/AgCl). ICP-OES was measured on a Shimadzu ICPE-9810 multitype ICP emission spectrometer. Samples for ICP-OES measurement were prepared by digesting samples in aqua regia.

### DOLFIN process

The experiments were performed in a clean room under yellow light. The 12 mm × 12 mm substrates (Si/SiO_2_, Si, quartz, glass) were treated by piranha (conc. H_2_SO_4_ and 30% H_2_O_2_, 7:3 v/v) followed by ultrapure water. Photoactive NC inks were passed through a 0.22 μm PTFE filter before use. In a general DOLFIN procedure, 20 μL of ILANs (25 mg mL^−1^ in DMF) mixed with PAmG-BTA was dropped onto the substrate and spin-coated (spread: 500 rpm, 10 s; spin: 2000 rpm, 60 s). The DUV light was provided by a low-pressure Hg lamp (254 nm, 3.6 mW cm^−2^). The film was exposed through a mask held together in a Mask Aligner system or by using binder clips. After exposure, the film was developed in DMF or NMF to remove unexposed ILANs and unreacted PAmG-BTA molecules. The exposure doses and related parameters for the patterning of NCs are listed in Table S4.

### Inkjet printing process

NCs modified by MPA or CA were dissolved in water mixed with 20% ethylene glycol (v/v). Ethylene glycol was added to control the viscosity and volatility of NC inks, which should be necessary to avoid possible nozzle clogging caused by solvent volatilization. The concentration can be 5–10 mg mL^−1^. The ink-jet printing was taken on dark parchment by using an HP DeskJet 2130 printer. To obtain brighter patterns, the printing process can be repeated several times.

### Reporting summary

Further information on research design is available in the Nature Portfolio Reporting Summary linked to this article.

## Supplementary information

Supplementary Information File

Reporting Summary

## Data Availability

The data that support the findings of this study are available from the corresponding author upon request.
