# Supplementary material for: Surface passivation of intensely luminescent all-inorganic nanocrystals and their direct optical patterning
Source: Nat Commun. 2023 Jan 4;14:49. doi: 10.1038/s41467-022-35702-7 (PMC9813348; doi:10.1038/s41467-022-35702-7)
Supplement: Supplementary file 1 — Supplementary Information File [file 41467_2022_35702_MOESM1_ESM.pdf]

Supplementary Information

Surface passivation of intensely luminescent all-inorganic nanocrystals and their direct optical patterning

Pengwei Xiao, Zhoufan Zhang, Junjun Ge, Yalei Deng, Xufeng Chen, Jianrong Zhang, Zhengtao Deng, Kambe Yu, Dmitri V. Talapin,* Yuanyuan Wang*

[1] *State Key Laboratory of Coordination Chemistry, School of Chemistry and Chemical Engineering, Nanjing University, Nanjing 210023, China*

[2] *Department of Chemistry and James Franck Institute, University of Chicago, Chicago, Illinois 60637, United States*

[3] *College of Engineering and Applied Sciences, Nanjing University, Nanjing, 210023, China*

[4] *NanoPattern Technologies, Inc., Chicago, Illinois 60637, United States*

* To whom correspondence should be addressed. E-mail: dvtalapin@uchicago.edu, wangyy@nju.edu.cn

SUPPLEMENTARY FIGURES

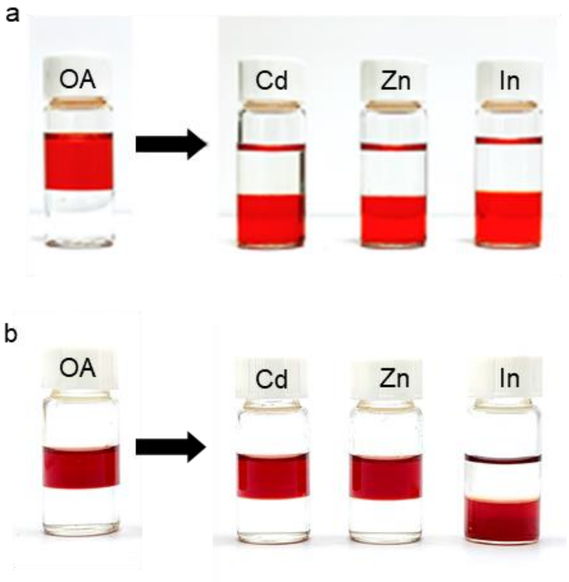

Supplementary Figure 1. Optical images of (a) CdSe NCs and (b) InP NCs before and after treatment with Cd^{2+}, Zn^{2+} and In^{3+} salts. The solvents of upper layer and bottom layer were hexane and DMF, respectively.

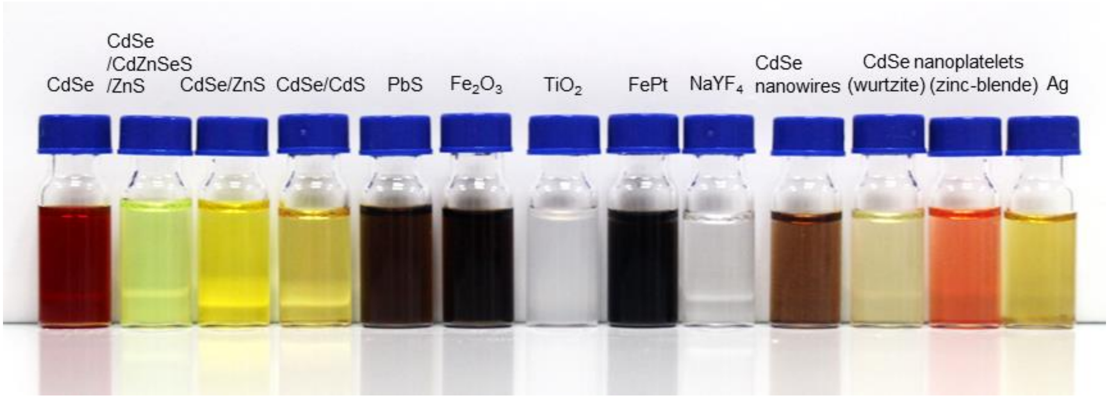

Supplementary Figure 2. Colloidal solutions of the all-inorganic NCs. From left to right: CdSe, CdSe/CdZnSeS/ZnS, CdSe/ZnS, CdSe/CdS, PbS, Fe_2O_3, TiO_2, FePt, $NaYF_4$, CdSe nanowires, CdSe nanoplatelets (wurtzite), CdSe nanoplatelets (zinc-blende), Ag NCs. $In(NO_3)_3$ treated: CdSe/CdZnSeS/ZnS, CdSe/ZnS; $In(BF_4)_3$ treated: PbS, Fe_2O_3, TiO_2, FePt, $NaYF_4$; $In(OTf)_3$ treated: CdSe nanowires, CdSe nanoplatelets (zinc-blende); $Zn(NO_3)_2$ treated: CdSe, CdSe/CdS; $Cd(NO_3)_2$ treated: CdSe nanoplatelets (wurtzite), Ag NCs.

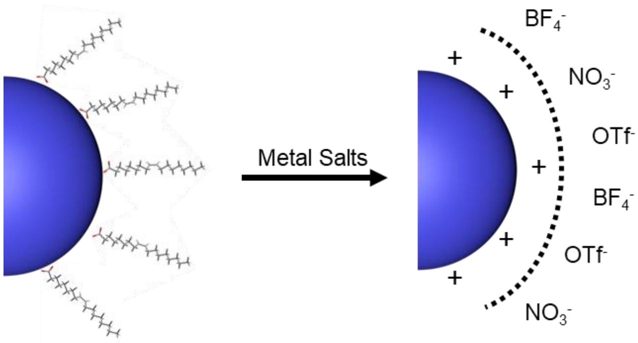

Supplementary Figure 3. Schemes of diffuse double layer around the all-inorganic nanocrystal surface. Non-coordinating anions (OTf^-, NO_3^-, BF_4^-) play a role in charge balance and stabilize the colloidal particles.

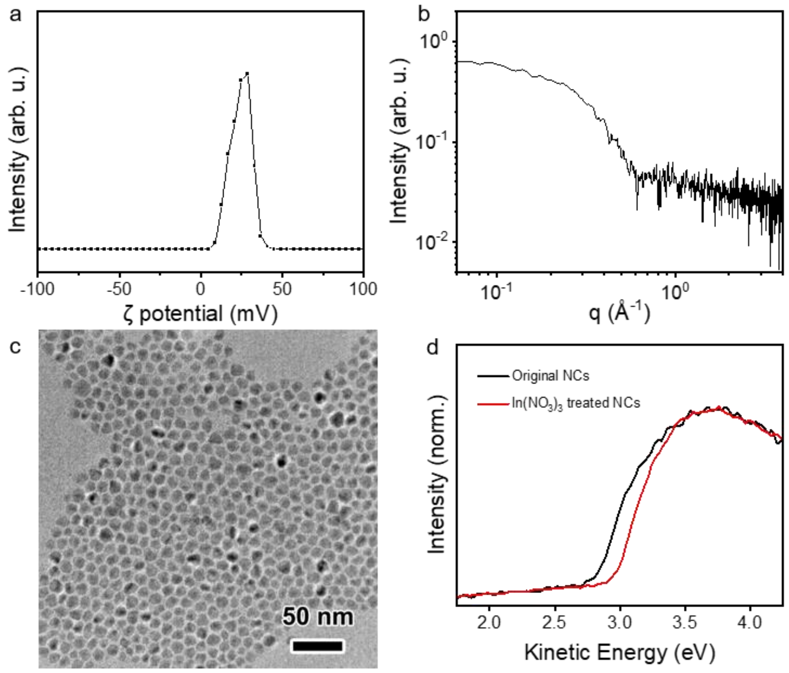

Supplementary Figure 4. Supplementary characterizations of ILANs. (a) ζ-potential measurement of $In(NO_3)_3$-treated CdSe/CdZnSeS/ZnS NCs. (b) SAXS graph of CdSe/CdZnSeS/ZnS ILANs in DMF. (c) TEM images of CdSe/CdZnSeS/ZnS NCs capped by OA. (d) UPS (secondary electron cutoff regions) of OA-capped NCs and $In(NO_3)_3$ treated CdSe/CdZnSeS/ZnS NCs.

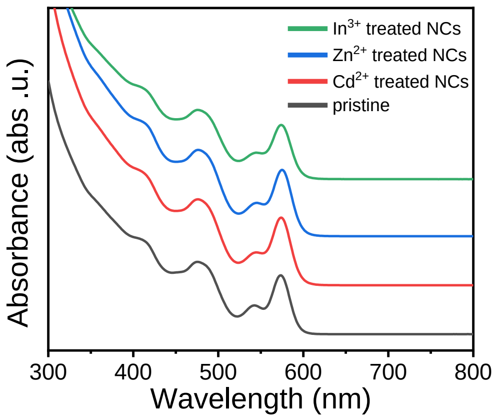

Supplementary Figure 5. Absorption spectra of CdSe NCs before and after treatment with Cd^{2+}, Zn^{2+} and In^{3+} salts. The signals at 573 nm, 542 nm and 475 nm belong to the first excitonic transition ($1S_{3/2}$-1Se) and other higher-energy transitions of 3.6-nm CdSe QDs.

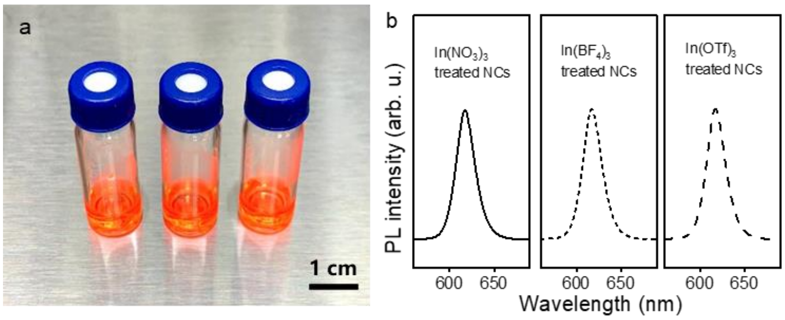

Supplementary Figure 6. Optical image (a) and PL spectra (b) of $In(NO_3)_3$, $In(BF_4)_3$ and $In(OTf)_3$ treated CdSe/ZnS NCs.

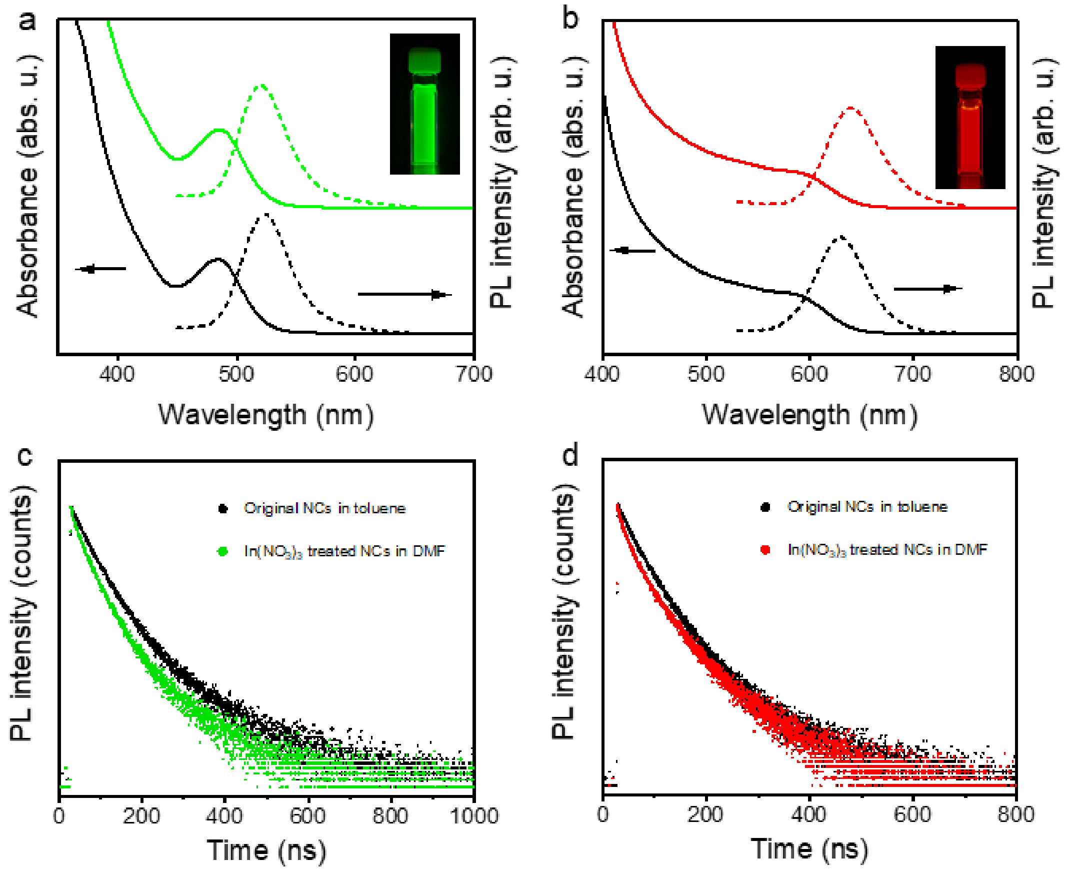

Supplementary Figure 7. Absorption, PL spectra (a, b) and transient PL kinetics (c, d) of green and red-emitting InP/ZnSeS/ZnS NCs before (black lines) and after (colorful lines) treatment with In(NO_3)$_3$.

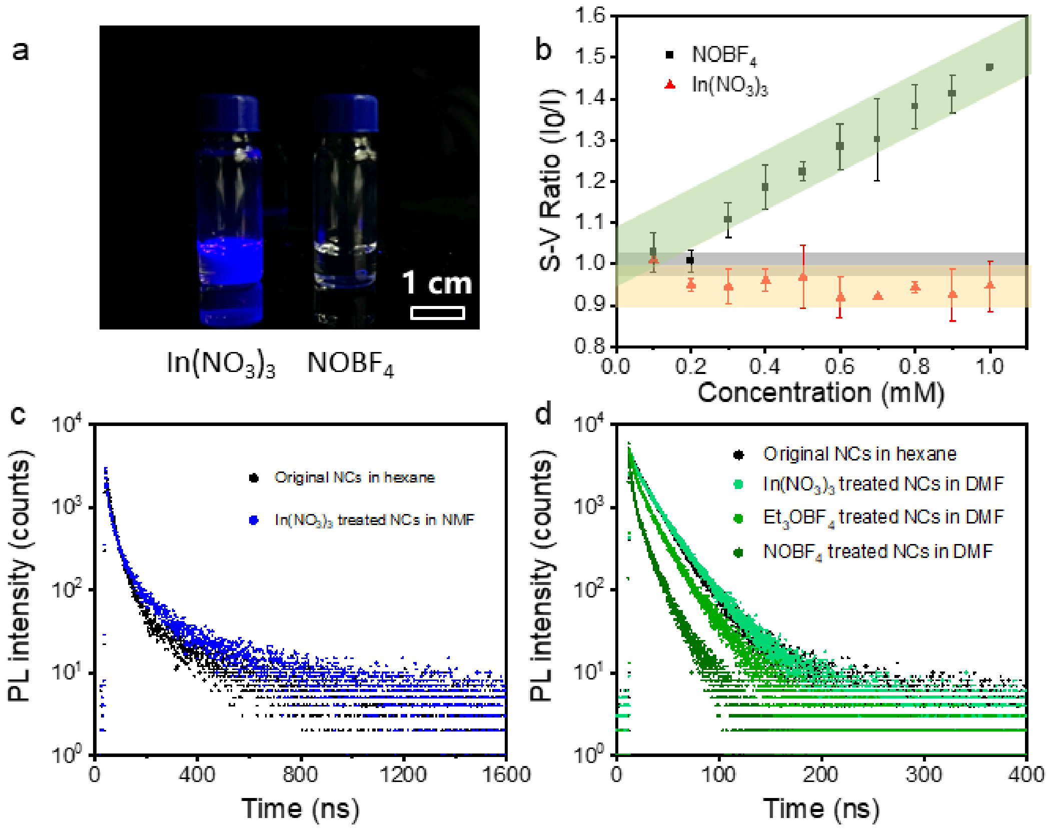

Supplementary Figure 8. Supplementary optical measurements of ILANs. (a) Optical image of blue-emitting NCs treated with $In(NO_3)_3$ and $NOBF_4$ (photographed under UV light). (b) Stern-Volmer ratio of CdSe/CdZnSeS/ZnS NCs treated with $NOBF_4$ and $In(NO_3)_3$ at concentrations from 0 to 1 mM. (c, d) Transient PL kinetics of blue and green NCs treated with different inorganic ligands.

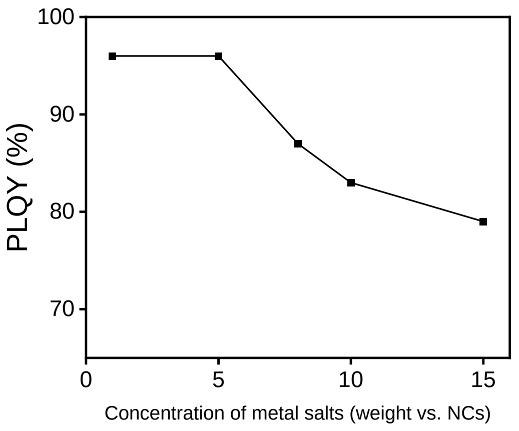

Supplementary Figure 9. The PLQYs of red-emitting CdSe/ZnS NCs treated by $In(NO_3)_3$ with various concentration.

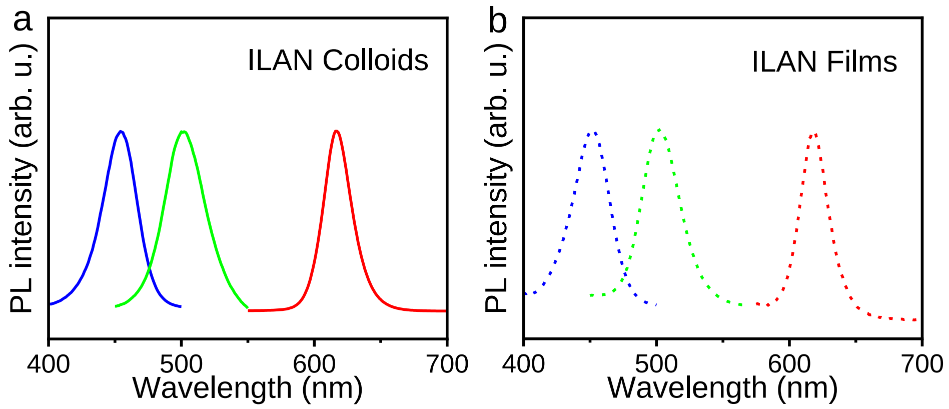

Supplementary Figure 10. PL spectra of blue (CdZnS/ZnS), green (CdSe/CdZnSeS/ZnS) and red-emitting (CdSe/ZnS) ILANs in the form of colloids (a) and films (b).

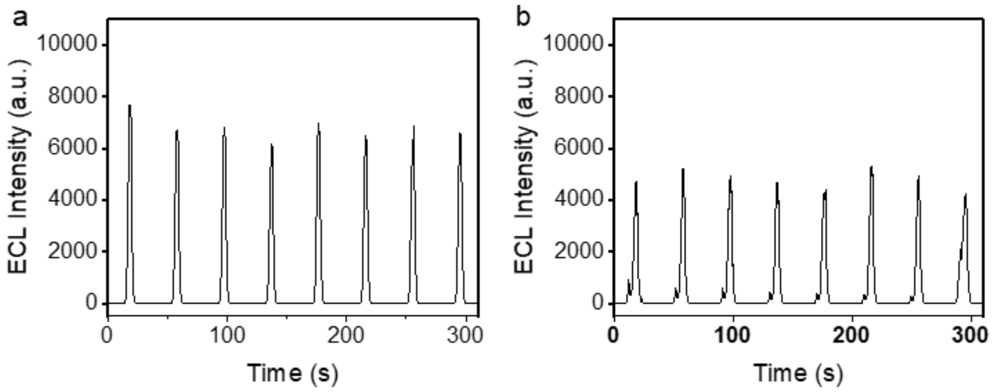

Supplementary Figure 11. ECL intensity of (a) organics-capped CdSe/ZnS NCs and (b) CdSe/ZnS ILANs.

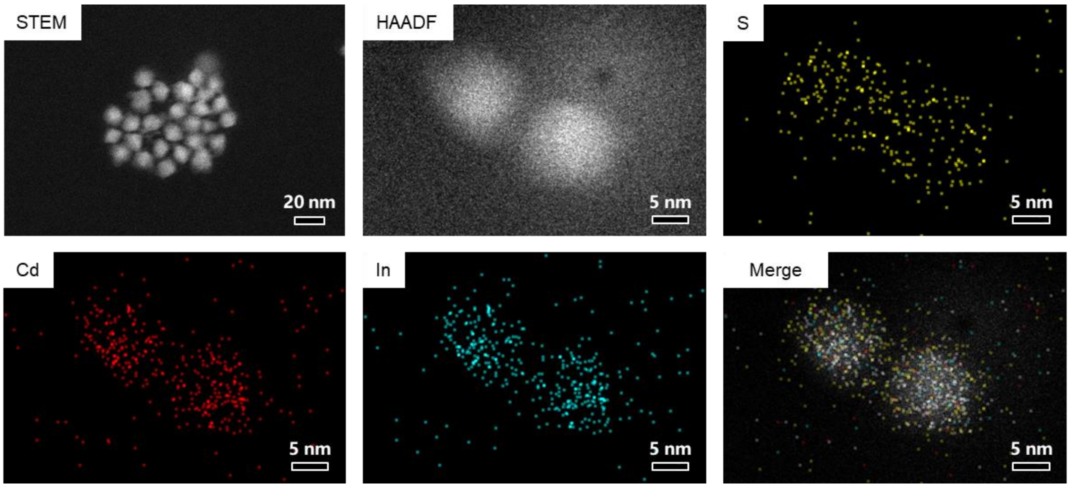

Supplementary Figure 12. Scanning transmission electron microscopy (STEM) image and energy dispersive spectroscopy (EDS) mapping images of $In(NO_3)_3$ treated CdSe/ZnS NCs.

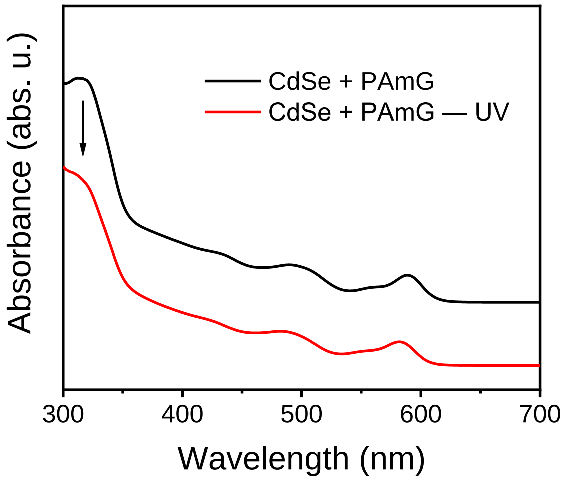

Supplementary Figure 13. Absorption spectra of CdSe NCs mixed with PAmG-BTA before (black line) and after (red line) photodecomposition.

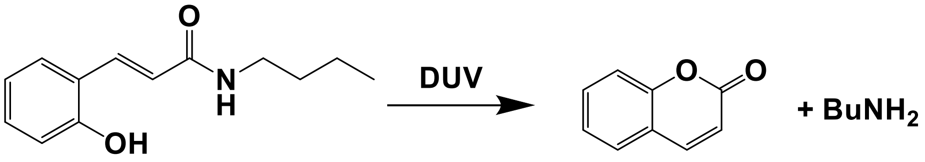

Supplementary Figure 14. Chemical equation of PamG-BTA photolysis.

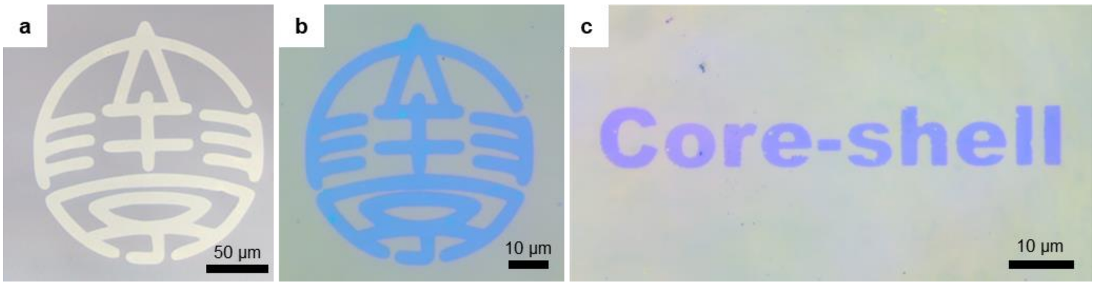

Supplementary Figure 15. Optical images of (a) PbS NC (b) ZnSe NC, and (c) InP/ZnS NC patterns via PAmG-assisted DOLFIN.

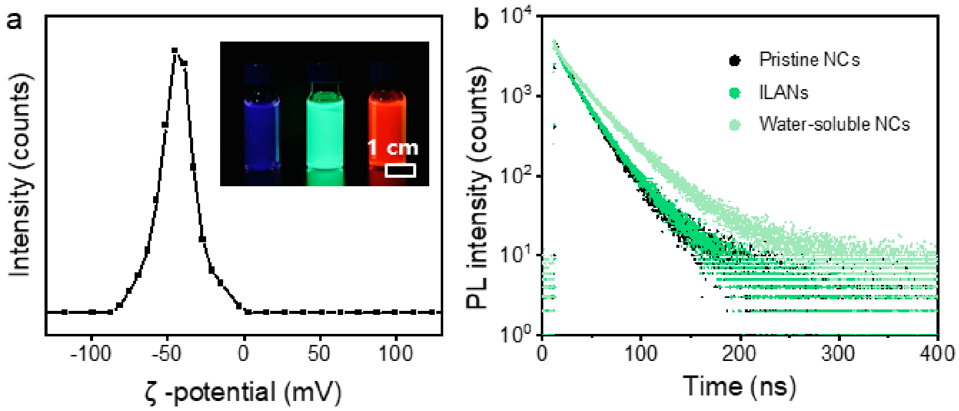

Supplementary Figure 16. Water-soluble NCs. (a) ζ-potential measurement of ILANs modified by MPA. Inset: optical image of the red, green and blue nanoinks. (b) PL decay curves of MPA-modified NCs compared with original and all-inorganic NCs. The PLQY of MPA-modified green-emitting CdSe/CdZnSeS/ZnS NCs was 91% in water, and the lifetime was 27 ns.

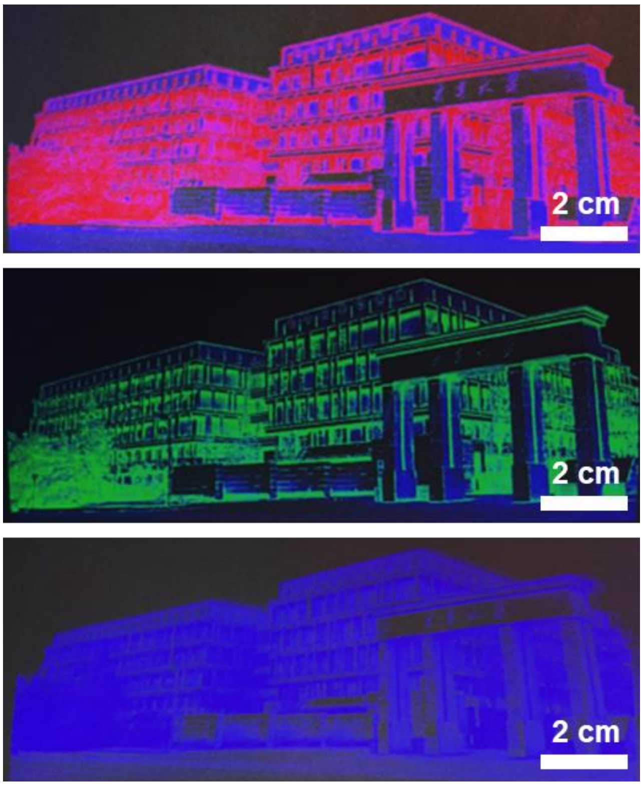

Supplementary Figure 17. Single-color ink-jet printing of blue, green and red NCs.

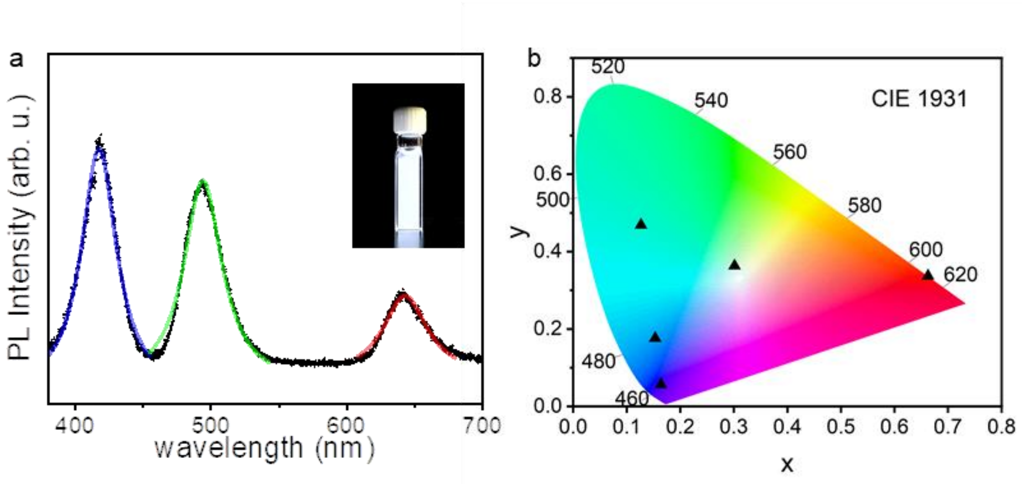

Supplementary Figure 18. The PL spectrum (a) and CIE coordinates (b) of the nanoinks. The three emission peaks can be fitted into red, green and blue features. Inset: optical image of white ink.

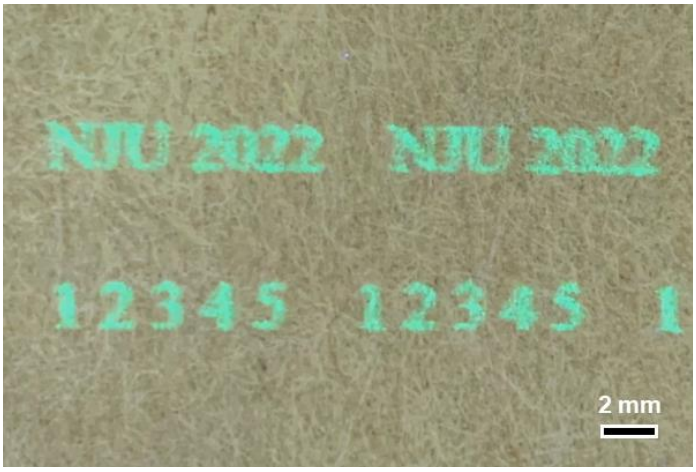

Supplementary Figure 19. Optical image of inkjet-printed patterns on parchment paper.+

SUPPLEMENTARY TABLES

Supplementary Table 1. QY and lifetime of NCs capped with OA and treated with $In(NO_3)_3$

NCs	OA		$In(NO_3)_3$	
	QY	Lifetime	QY	Lifetime
CdSe/ZnS (red)	97%	24 ns	97%	24 ns
CdSe/CdZnSeS/ZnS (green)	84%	18 ns	80%	18 ns
CdZnS/ZnS (blue)	82%	38 ns	72%	34 ns
InP/ZnSeS/ZnS (red)	66%	45 ns	39%	40 ns
InP/ZnSeS/ZnS (green)	76%	64 ns	57%	48 ns

Supplementary Table 2. Film PLQY of pristine NCs and ILANs

	Film PLQY	
	Pristine	ILANs
CdSe/ZnS (red)	80%	75%
CdSe/CdZnSeS/ZnS (green)	76%	68%
CdZnS/ZnS (blue)	78%	58%

Supplementary Table 3. CdSe/ZnS ILAN Film thickness depended on ink concentration and spin-coating parameter

Ink concentration	Spin-coating parameter	Thickness
50 mg/mL	2000rpm 60s	65±4 nm
30 mg/mL	2000rpm 60s	54±6 nm
30 mg/mL	3000rpm 60s	46±6 nm
15 mg/mL	3000rpm 60s	38±4 nm
15 mg/mL	5000rpm 60s	23±4 nm

Supplementary Table 4. Patterning conditions of PAmG-assisted DOLFIN

NCs	Concentration (mg/mL)	PAmG-BTA (wt, vs. NCs)	Ink Solvent	Developers	Dose (254 nm, mJ/cm^2)
CdSe	20	0.2	DMF	DMF, NMF	45-120
ZnSe	20	0.2	DMF	DMF, NMF	45-90
PbS	20-30	0.2	DMF	DMF, NMF	45-90
CdSe/ZnS	20-30	0.4	DMF	NMF	80-110
CdSe/CdZnSeS/ZnS	20-30	0.4	DMF	NMF	80-110
CdZnS/ZnS	20-30	0.4	DMF	NMF	80-110
InP/ZnS	20	0.4	DMF	NMF	80-110
